# Change in lumbar lordosis during prone lying knee flexion test in subjects with and without low back pain

**DOI:** 10.1186/s12998-015-0061-z

**Published:** 2015-06-01

**Authors:** Amir M Arab, Ailin Talimkhani, Noureddin Karimi, Fetemeh Ehsani

**Affiliations:** Department of Physical Therapy, University of Social Welfare and Rehabilitation Sciences, Velenjak, Tehran, Iran; University of Social Welfare and Rehabilitation Sciences, Velenjak, Tehran, Iran

**Keywords:** Low back pain, Lumbar lordosis, Movement pattern, Prone knee flexion, Flexible ruler

## Abstract

**Background:**

Prone lying knee flexion (PLKF) is one of the clinical tests used for assessment of the lumbo-pelvic movement pattern. Considerable increase in lumbar lordosis during this test has been considered as impairment of movement patterns in lumbar-pelvic region. However, no study has directly evaluated the change in lordosis during active PLKF test in subjects with low back pain (LBP). The purpose of this study was to investigate the change of lumbar lordosis in PLKF test in subjects with and without LBP.

**Methods:**

A convenience sample of 80 subjects participated in the study. Subjects were categorized into two groups: those with chronic non-specific LBP (N = 40, mean age: 40.84 ± 17.59) and with no history of LBP (N = 40, mean age: 23.57 ± 10.61). Lumbar lordosis was measured with flexible ruler, first in prone position and then on active PKF test in both subjects with and without LBP. Data was analyzed by using statistical methods such as, independent t-test and paired t-test.

**Results:**

There were statistically significant differences in lumbar lordosis between prone position and after active PLKF in both subjects with and without LBP (P < 0.0001). The amount of change in lordosis during PLKF test was not significant between the two groups (P = 0.65). However these changes were greater among patients with LBP.

**Conclusion:**

Increase in lumbar lordosis during this test may be due to excessive flexibility of movement of the lumbar spine in the direction of extension and abnormal movement patterns in the individuals with LBP.

## Background

Low back pain (LBP) is a world-wide health problem and the most common and costly musculoskeletal disorder in the today’s societies [1,2]. The prevalence of LBP is estimated to be between 10% and 80% depending on the population [3,4]. Despite its high prevalence and detrimental effects on subjects’ activities, the exact causes of mechanical LBP have not yet been fully understood. However, during the past decades the approach in evaluation and management of LBP has been changed from strengthening or stretching of the lumbo-pelvic muscles toward modification of the motor system and movement pattern [5].

A balanced motor system is obtained from coordinated activity of synergist and antagonist muscles. Normal functioning of the trunk depends not only on passive joint mobility, but also on normal muscular activity and central nervous system regulation. Muscles produce and control the movement and stabilize the spine, protecting if from excessive load during functional activities [6,7].

With regard to this point of view, repetitive movements and long-term faulty postures and movements can change muscle tissue characteristics and can lead to muscle dysfunction, altered movement pattern, pain and finally movement disorders [5]. Hence, the main emphasis has been recently placed on assessment of the altered movement pattern in patients with musculoskeletal pain and disorders such as LBP and on the important of achieving normal pattern of the movement for the prevention and treatment of LBP [6-11].

Several studies have demonstrated that LBP is associated with muscle imbalance and altered activation pattern of the lumbo-pelvic muscles during different tasks [12-15]. Some clinical tests have been used to assess the altered movement pattern in subjects with musculoskeletal disorders. Prone lying knee flexion (PLKF) is an accepted test for assessment and treatment of the lumbo-pelvic movement patterns [5]. In this test, a patient lays prone and actively flexes his or her dominant knee as far as possible. Muscle imbalance and altered activation of the lumbo-pelvic muscles has been reported during PLKF test in patients with chronic LBP [5]. Excessive anterior pelvic tilt, lumbar rotation, lumbar hyperextension, increased lumbar lordosis and decreased knee flexion during the PLKF has been considered as abnormal movement patterns during PLKF [5]. Coordination between muscles in the lumbo-pelvic region is thought to balance the position of the pelvis in normal posture and during the lower limb or trunk movement. It has been assumed that during PLKF, lack of sufficient stiffness in the abdominal and anterior supporting structures of the lumbar spine produces anterior tilt in the pelvic and increased lumbar lordosis specially in person with lower cross syndrome [5].

However, to our knowledge, no study has investigated the change in lumbar lordosis during PLKF in patients with chronic LBP. The purpose of this study was to investigate the change in the degree of lumbar lordosis during PLKF in subjects with and without chronic LBP and to determine if this change varies between two groups.

## Methods

### Subjects

The quasi-experimental study design with repeated measurements was used to investigate the lumbar lordosis changes during PLKF in two groups: subjects with chronic non-specific LBP (N = 40, average age: 40.84 [SD = 17.59] years old, average height: 165.0 [SD = 9.0] cm, average weight: 70.31 [SD = 16.06] kg, body mass index (BMI): 25.55 [SD = 3.99] kg/m^2^) and subjects with no history of LBP (N = 40, average age: 23.57 [SD = 10.61] years old, average height: 162.0 [SD = 7.0] cm, average weight: 55.62 [SD = 6.55] kg, BMI: 21.05 [SD = 2.26] kg/m^2^).

Power analysis was used to determine the sample size for test. Type I error (α) was set at 0.05 and power of the test was 0.80. Considering this, the calculated sample size showed that sample size in this study was appropriate to test the hypothesis and the results derived from the study are meaningful.

The subject population in this study was a sample of convenience. The LBP patients were referred by orthopedic specialist and physiotherapy clinics. The patients were included if they had a history of non-specific LBP for more than six weeks duration before the study date. They were also included if had intermittent (on and off) LBP with at least three previous episodes each lasting more than one week, during the year before the study [16].

The control group was evaluated and found to have no complaint of any pain or dysfunction in their low back, pelvis, thoracic and lower extremities. The healthy subjects were recruited from the university students.

The exclusion criteria in both groups were pregnancy, history of dyspnea, history of hip pain, dislocation or fracture, history of lumbar spine surgeries, history of anterior knee ligament injury or rupture, history of anterior knee pain, inability to perform active PLKF without pain, history of lower extremity injury in the past 3 months, shortness of hip flexors, positive neurological symptoms and cardiopulmonary disorders. Each eligible subject was enrolled after signing an informed consent form approved by the human subjects committee at the University of Social Welfare and Rehabilitation Sciences. Ethical approval for this study was granted from the internal ethics committee at the University of Social Welfare and Rehabilitation Sciences (Date: 2013.03.09).

### Procedures

The subject was on the examining table in the prone position. The lumbar lordosis was measured first in prone position. Then the subject was asked to perform knee flexion (PLKF) and then the lumbar lordosis was measured after PLKF test in both subjects with and without LBP. The dominant leg was chosen for investigation.

### Measuring lumbar lordosis

**Figure 1:**
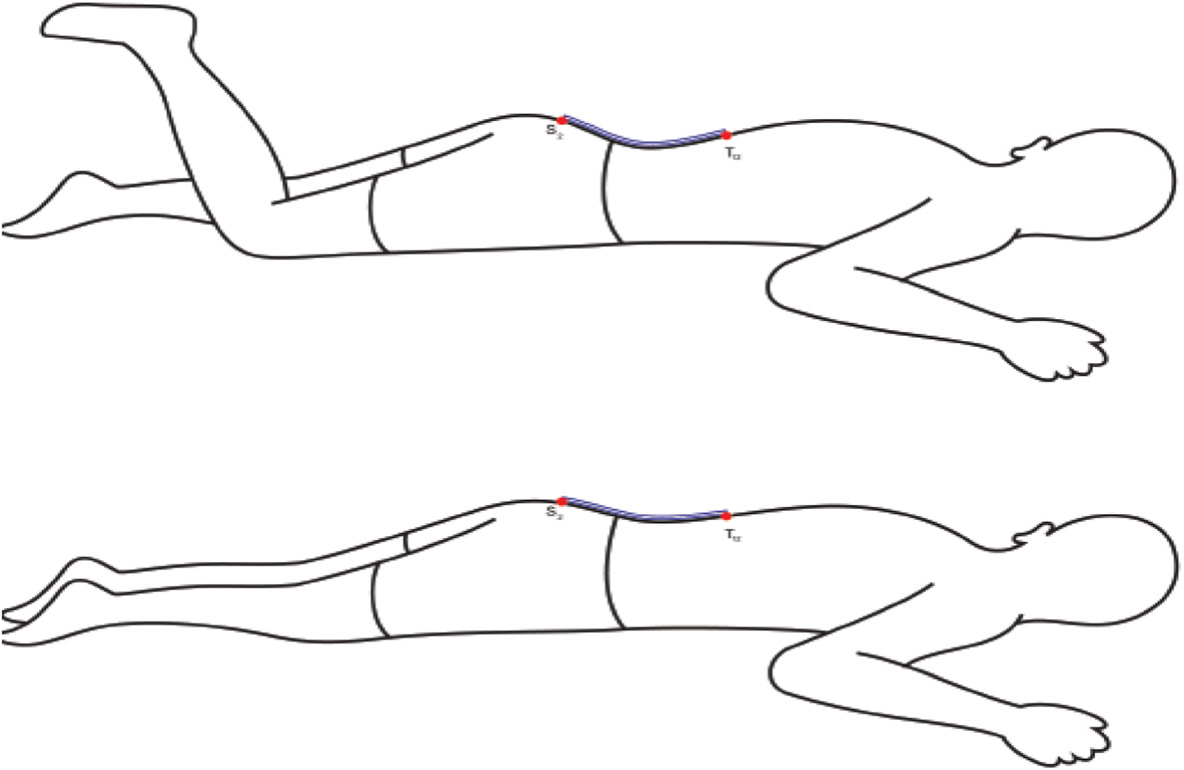
Measurement of lumbar lordosis with flexible ruler in prone position and active PLKF.

A standard flexible ruler was used to measure the degree of lumbar lordosis in prone position before and after active knee flexion (Figure 1). For this purpose, the subject’s position was prone lying on a treatment table with the arms along the sides and head face was down. The base of sacrum and spinous process of L1 was located by palpation and marked with removable stickers.

A standard flexible ruler was fitted on subject’s lumbar curve, over the lumbar spinous processes of L1 – S1. The curve of the flexible ruler, resembling the size of subject’s lumbar curvature, was graphed on a paper, noting where the two reference points for L1 and S1 were located. The method explained by others was used to quantify the degree of lumbar lordosis [16-20].

Two points on the curve, representing L1 and S1, were connected by a line (L). A perpendicular line (H), representing the height of the lumbar curve, bisected line L. The length of each line was calculated in millimeters, and the values were used in the following formula to calculate the degree of lumbar lordosis.

$$ \uptheta = 4\ \left[\mathrm{Arc}\  \tan\ \left(2\mathrm{H}/\mathrm{L}\right)\right] $$

A very high correlation (r = 0.92) has been found between degrees of lumbar lordosis measured by a flexible ruler and from lumbar X-rays [21-23]. The reliability of flexible curve for measurement of lumbar lordosis has been previously established [24].

### Data analysis

Statistical analysis was performed using SPSS version 16.0.

A paired *t*-test was used to demonstrate changes in lumbar lordosis before and after PLKF test in both subjects with and without LBP.

An independent *t*-test was used to compare changes in lumbar lordosis during PLKF between subjects with and without LBP and also to compare demographic data between subjects with and without LBP. Statistical significance was attributed to P value less than 0.05.

### Ethical approval

This research was reviewed and was approved by the Human Subject Committee at University of Social Welfare and Rehabilitation Sciences.

## Results

The demographic data for the subjects are presented in Table 1. No statistical significance was found in the height between groups. However, there was a statistically significant difference in subjects’ age, weight and BMI between the two groups (P = 0.000).

**Table 1:** **Demographic data of the subjects in each group**

**Variables**	**With no LBP (n = 40)**	**With LBP (n = 40)**
Age (years)	23.57 (10.61)	40.84 (17.59)
Weight (kg)	55.62 (6.55)	70.31 (16.06)
Height (cm)	162.0 (7.0)	165.0 (9.0)
BMI (kg/m^2^)	21.05 (2.26)	25.55 (3.99)

Continuous data: Mean (Standard Deviation).

LBP = Low Back Pain, BMI = Body Mass Index.

**Figure 2:**
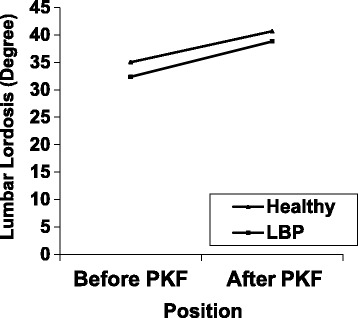
Lumbar lordosis during PLKF in subjects with and without LBP.

There was no significant difference in lumbar lordosis at the baseline in prone relaxed position between two groups (P = 0.21, %95 CI: 1.77-7.44). There was a statistically significant difference in lumbar lordosis between prone position and after PLKF in subjects without LBP (P = 0.000) and subjects with LBP (P = 0.000) (Table 2, Figure 2). Overall, the lumbar lordosis was significantly greater in the PLKF compared to prone-relaxed position in both subjects with and without LBP. The mean difference in lumbar lordosis between positions was 6.47 and 5.65 for subjects with LBP and without LBP respectively.

**Table 2:** **Lumbar lordosis in both groups**

**Variables**	**Before PLKF**	**After PLKF**	**P-value**
With no LBP	35.02 (9.25)	40.67 (13.09)	**0.000**
With LBP	32.35 (12.43)	38.82 (14.43)	**0.000**

Continuous data: Mean (Standard Deviation). Bold p-values indicate statistical significance.

LBP = Low Back Pain, PLKF = Prone Lying Knee Flexion.

There was no statistically significant difference in the changes of lumbar lordosis after performing PLKF between subjects with and without LBP (P = 0.65) (Table 3). However, the changes in lumbar lordosis were greater among patients with LBP compared to those without LBP.

**Table 3:** **Changes in lumbar lordosis between two groups**

**Variable**	**With no LBP (n = 40)**	**With LBP (n = 40)**	**P-value**
Changes in lumbar lordosis during PLKF	5.65 (10.13)	6.47 (6.67)	0.65

Continuous data: Mean (Standard Deviation).

LBP = Low Back Pain, PLKF = Prone Lying Knee Flexion.

## Discussion

The current study shows changes in lumbar lordosis during active PLKF test in subjects with and without LBP. The results of this study demonstrated that there were statistically significant differences in lumbar lordosis between prone position and after PLKF in both subjects with and without LBP (P < 0.0001). But the amount of changes in lordosis during PLKF test was not significant between two groups (P = 0.65). These changes were greater among patients with LBP compared to subjects without LBP.

In this study, the subjects had no pain during the test and none of the subjects reported that pain was a limiting factor to perform PLKF test, so, direct effects of pain on the measurement can be minimized.

Lumbar extension and anterior rotation of the pelvis are often observed during the PLKF test. Increase in the degree of lumbar lordosis during PLKF test found in both groups can be attributed to the accompanied lumbar extension during flexion of the knee. In theory, it is proposed that excessive anterior pelvic tilt, lumbar hyperextension and increased lumbar lordosis during the PLKF are commonly seen as abnormal movement patterns in patients with chronic LBP [5]. Investigators attributed these to muscle imbalance and altered activation of the lumbo-pelvic muscles [5].

Previous investigators attributed excessive lumbar extension and hyper lordosis during PLKF to a deficiency in controlling anterior pelvic rotation during PLKF because of muscular dysfunction in the lumbo-pelvic region [5,25]. Sahrmann [5] proposed the concept of “relative flexibility or stiffness” that has been linked to uncontrolled movement, pain and pathology by causing direction related stress and strain during various functional movements in the patients with LBP. Sahrmann [5] suggested that increased stiffness of the anterior supporting structures of the thigh, hip, knee and lumbar spine can result in compensatory exaggerated anterior pelvic tilt with lumbar extension motion during prone knee flexion or hip extension. In this study, stiffness in thigh and anterior supporting structures of the lumbar spine was not measured, just measured the change in lumbar lordosis during PLKF.

Scholtes et al. [26] found that during knee flexion and hip lateral rotation in prone lying, subjects with LBP demonstrated a greater maximal lumbar-pelvic rotation angle compared to those without LBP, as the lumbar-pelvic region may move more frequently during the early ranges of lower limb movement in daily activities.

In this study, lumbar lordosis was significantly higher during PLKF compared to prone relaxed position in both subjects with and without LBP. However, this change in lumbar lordosis during PLKF was not significant between two groups. The reason for this may be due to the healthy subjects being recruited from university students and staff used to performing sustained postures and repeated movements in their daily activities.

It has been thought that, if the lumbar-pelvic motion occurs more during a limb movement, then the frequency of lumbar-pelvic motion may be increased through the day. The increased frequency of the movements in lumbar-pelvic region can contribute to increased mechanical stress and strain on lumbar-pelvic region. This can also change the characteristics of muscular tissue, in turn, leading to abnormal movement patterns in lumbar-pelvic region [26,27]. Previous studies supported increased mobility of the lumbar-pelvic region in LBP patients which can be associated with degeneration of lumbar-pelvic region tissues [28,29].

In this study, compensatory lumbar extension motion during active PLKF test may be due to instability in lumbar-pelvic region and also, excessive flexibility of movement of the lumbar spine in the direction of extension. This hypothesis has been supported by findings which suggest active limb movements which contribute to accumulation of tissue stress can affect decrease in spinal stability in patients with LBP [30]. However, more studies are needed to resolve the existing ambiguities in this field.

### Limitations

We acknowledge some limitations. In this study the patients with chronic non-specific LBP were examined and other LBP patients (acute or specific LBP) were not examined. Another limitation of this study was that LBP subjects were not categorized based on movement system impairment-based categories for LBP as described by Sahrmann [5].

It has been suggested investigating the lumbar lordosis change in LBP patients with different movement system impairment-based categories. Again in this study, we did not measure lumbar -pelvic kinematics and electromyography (EMG) activity of the stabilizing and prime mover muscles during PLKF to find the pattern of muscles recruitment.

The fact that the healthy subjects were recruited from university students and staff performing sustained postures and repeated movements in their daily activities may be used to question the results showing no significant difference in lumbar lordosis change during PLKF between two groups.

Considering the non statistically different but measurable changes in lumbar lodosis during PLKF between subjects with and without LBP, we suggest that PLKF can be used as an evaluation tool of lumbar-pelvic movement patterns in the individuals with LBP and even healthy individuals with poor postural alignment and poor movement habits.

## Conclusion

This study investigated the chansge in lumbar lordosis during PLKF test between subjects with and without LBP. The results of this study indicate an increase in the degree of lumbar lordosis during PLKF compared to prone-relaxed position in subjects with and without LBP. However, greater change in lumbar lordosis was found in the subjects with LBP compared to healthy subjects. More studies are needed to resolve the existing ambiguities in this field.
